# Psychological needs, self-efficacy, motivation, and resistance training outcomes in a 16-week barbell training program for adults

**DOI:** 10.3389/fpsyg.2024.1439431

**Published:** 2024-09-02

**Authors:** Vanessa M. Martinez Kercher, Janette M. Watkins, Janelle M. Goss, Liam A. Phillips, Brad A. Roy, Kyler Blades, Dana Dobson, Kyle A. Kercher

**Affiliations:** ^1^Department of Health & Wellness Design, School of Public Health-Bloomington, Indiana University, Bloomington, IN, United States; ^2^Department of Kinesiology, School of Public Health-Bloomington, Indiana University, Bloomington, IN, United States; ^3^Program in Neuroscience, College of Arts and Sciences, Indiana University, Bloomington, IN, United States; ^4^United States Military Academy, Westpoint, NY, United States; ^5^Logan Health Medical Center, Kalispell, MT, United States; ^6^Blades Athletic Performance Academy, Kalispell, MT, United States

**Keywords:** strength training, affective responses, barbell training, women's health, inclusion

## Abstract

**Background:**

Despite extensive research on the relationship between psychological factors and aerobic training, there remains a gap in understanding these relationships within resistance training (RT), particularly barbell-based RT. This study aimed to examine the associations between basic psychological needs, behavioral regulation, self-efficacy, and a longitudinal barbell-based RT program for adults.

**Methods:**

Forty-three adults (*M* age = 45.09 ± 10.72) were recruited from the Competitive Edge resistance training program at a medical fitness center in Northwest Montana. The study followed an 18-week schedule: 8 weeks of training, 1 week of active recovery, and 8 additional weeks of training.

**Results:**

The results reveal several significant findings. First, the basic psychological need for competence significantly increased from baseline (*M* = 5.06) to post-program (*M* = 5.30), (*p* = 0.017). Second, the composite score of the BREQ-3 significantly predicting muscular strength improvements in the deadlift (*β* = 3.64, *p* = 0.039). Third, both mastery (*p* = 0.021) and resilience (*p* = 0.007) self-efficacy subscales increased from baseline to post-program. Fourth, exploratory analyses indicated that the reasons to exercise scale predicted increases in muscular endurance with the weight management (*β* = 10.016, *p* = 0.046) and solitude (*β* = 6.792, *p* = 0.037) subscales.

**Conclusion:**

These findings highlight the importance of psychological factors in predicting strength outcomes and muscular endurance, suggesting that psychological interventions may complement physical training to maximize benefits. This research contributes valuable insights into how psychological factors influence training outcomes, potentially guiding future interventions and program designs to better support strength development and endurance in resistance training contexts.

## Introduction

Resistance training (RT) is a crucial form of exercise for adults of all ages, with training benefits observed among diverse populations (Centers for Disease Control and Prevention, 2006). It is even backed by public health agencies, with the Centers for Disease Control (CDC) advising that individuals aged 18–64 engage in muscle-strengthening activities at least twice a week. Despite its significance for promoting healthy adulthood, RT experiences less emphasis, adherence, and popularity compared to aerobic training. Only 31% of adults meet the recommended guidelines for strength training (Elgaddal et al., 2022). Among this 31%, only 6.8% adhere exclusively to strength training guidelines, while 24.2% meet both aerobic and anaerobic training guidelines. Furthermore, despite the importance of psychological factors to RT initiation, improvements, and adherence, there is a lack of multidisciplinary studies assessing the relationship between RT and psychological factors. Rather, the studies that exist regarding psychology and RT typically only assess single joint exercises, single sessions, machine-based RT exercises (e.g., bicep curls), or competitive athletes. To our knowledge, this is the first study to examine basic psychological needs, self-efficacy, reasons for exercises, and RT strength improvements in adults enrolled in a longitudinal RT program.

Importantly, women are significantly underrepresented in exercise science research. A recent systematic review found that only 8.8% of studies concentrate on women compared to 70.7% focusing on men, with just 20.5% of studies including both genders (Paul et al., 2023). These research deficits translate to observed behaviors, with only 20% of women reporting participation in RT (Centers for Disease Control and Prevention, 2006). A recent study revealed a striking contrast, indicating that for every woman using free weights in a gym, there were 27 men doing the same (a 27 to 1 ratio) (Haines et al., 2008). The American College of Sports Medicine identifies the perception of time and effort required as a significant barrier to resistance training among female adults (Hurley et al., 2018). Gender dimorphisms exist in motivation to strength train as well. Men have unique motivations for strength training, including physical health, sport or performance goals, physical appearance, and social factors (Ashton et al., 2015). Key motivators for men also encompass concerns about physical appearance, social inclusion, health benefits, and sport/performance improvements (Ashton et al., 2015). Women, however, may experience more potentially negative motivators, such as avoiding criticism from healthcare or fitness professionals, or following trends in social media or pop culture (Vasudevan and Ford, 2022). Additionally, women are often likely to be motivated by objectives centered on enhancing physical appearance, attractiveness, achieving muscle toning, and managing weight through weight loss or weight management approaches (Nuzzo, 2023). Importantly, in adults, valuing a variety of goals can lead to better self-motivation and higher levels of physical activity (Martinez Kercher et al., 2022). More specifically, research underscores the significance of aligning exercise with personal values and the supportive role of social environments in maintaining physical activity levels (Lev-Arey et al., 2024).

Participation in strength training is influenced by psychological factors such as self-efficacy and psychological needs. Self-efficacy is defined as an individual’s belief about their capabilities that can either aid or hinder exercise behaviors (Bandura, 1997). It can be influenced through successfully completing tasks (i.e., mastery experiences/performance accomplishments using tracking logs), hearing and seeing others’ experiences and successful application of strategies (i.e., modeling/vicarious experiences), social persuasion (i.e., having other individuals tell you that you can adopt the behavior), and reduction of stress and physical/emotional arousal (i.e., self-pacing activity that challenges one without creating anxiety) through education on technique. Research shows that self-efficacy, composed of measures of mastery, resilience, and physical ability, mediates the relationship between mindset and exercise frequency (Orvidas et al., 2018), suggesting that self-efficacy is important for exercise adherence in adults (Rhodes et al., 2017).

Self Determination Theory (SDT) may be a useful tool to analyze the effects of self-efficacy and other psychological factors on exercise behavior (Teixeira et al., 2012). SDT highlights three fundamental psychological needs: competence, autonomy, and relatedness (Ryan and Deci, 2000). Autonomy refers to self-direction and freedom in decision-making. Competence is about feeling capable and effective in tasks. Relatedness involves meaningful social connections and a sense of belonging. When these needs are met, individuals experience heightened self-motivation and improved mental health (Ryan and Deci, 2000). Likewise, factors such as autonomy and competence in strength training promote adherence (Martinez Kercher et al., 2022). Conversely, unmet needs can lead to reduced motivation and diminished well-being, including decreased/lack of participation in exercise (Martinez Kercher et al., 2022). In comparison, with the large amount of literature regarding aerobic exercise, relatively few studies have examined the relationship between evidence-based psychological factors and RT outcomes. To our knowledge, this is the first study to examine psychological needs for exercise, motivation to exercise, and RT outcomes in a longitudinal study of general population adults using compound barbell exercises. Furthermore, a strength of this study is the deliberate recruitment and inclusion of women in RT research.

The overarching goal of this study was to examine the relationship between psychological needs, behavioral regulation, self-efficacy, and a longitudinal barbell-based RT program for adults. This study had three objectives. The primary objective was to explore the longitudinal influence of 16-weeks of RT on psychological factors (e.g., basic psychological needs, self-efficacy, behavioral regulation in exercise) from baseline to post-program. We hypothesized that psychological factors would increase post-program compared to baseline. The secondary objective was to assess the influence of psychological factors on muscular strength and muscular endurance. We hypothesized that more positive psychological factors would predict greater gains in muscular strength and endurance testing outcomes. Finally, the exploratory objective was to assess the reasons for exercise (REx) scale. To do this, we tested if there were changes in reasons to exercise from baseline to post program, and if any REx subscales predicted changes in muscular strength and endurance testing outcomes.

## Methods

### Sample and setting

Participants were recruited from a RT program called Competitive Edge (CE), at an 8,000+ member medical fitness center in Northwest Montana. RT sessions took place in a semi-private turf area within the medical fitness center. 68% of the participants did not have a history of playing sports and were classified as novices/beginners (Haff and Triplett, 2016) based on previous RT experience. A convenience sample of 43 adults (*M* age = 45.09 ± 10.72) enrolled in Competitive Edge including 81.8% females (*n* = 35) and 18.2% males (*n* = 8) were recruited into this quasi-experimental study using a pretest-posttest design. 36 adults completed the 16-week study.

### Design

The study was conducted over the course of 18 weeks. The week 0 pre-screening assessments were followed by 8 weeks of training, 1 week of suggested active recovery at week nine, then another 8 weeks of training. For clarity, we designated the initial 8 weeks of training as weeks one through eight, and the subsequent 8 weeks as weeks nine through sixteen.

### Program

The Competitive Edge barbell program consisted of professionally supervised exercise, self-selected loads, and a small group atmosphere. The Competitive Edge barbell approach accommodated participants of any skill level. RT programming and protocols followed recommendations from the National Strength and Conditioning Association (NSCA) for optimal athletic development (Haff and Triplett, 2016). Coaches were certified by either NSCA, USA Weightlifting, and/or the American College of Sports Medicine. The classes were structured in an 8-week session with clients attending 2–3 classes per week. Each training group consisted of 4–9 participants. RT sessions lasted for approximately one-hour. Within each 8-week session there were two 4-week phases, each emphasizing muscular hypertrophy or strength development. We changed the phase every 4 weeks in accordance with NSCA-recommended phase principles and to avoid the ‘honeymoon’ effect (Rodgers et al., 2009). Within each 4-week phase there were three workouts: Day 1, Day 2, and Day 3 (see Supplemental File 1 for sample 4-week phase). Clients were encouraged to attend at least two workouts per week (either Mon, Wed or Tue, Thu) with an additional Day 3 workout also advised for Fridays or Saturdays. RT sessions included the following: (1) a 5–10 min warm-up consisting of various dynamic movements; (2) a muscle activation/coordination exercise; (3) a compound power exercise typically using barbell (e.g., hang clean); (4) a compound strength-based exercise typically using a barbell (e.g., squat); (5) multiple supporting exercises; (6) a challenging and camaraderie-building exercise called a “finisher.” These warm-ups were different each of the 3 days and changed each phase to avoid monotony.

### Measures

#### Physical activity, demographics, resistance training background questionnaire (PADRTBQ)

Participants reported their age, gender, ethnicity, and resistance training background using the Physical Activity, Demographics, and Resistance Training Background Questionnaire (PADRTBQ). Participants were asked about their exercise-related habits, such as resistance training history, time elapsed from starting with barbell training (in months), weekly frequency of barbell training, types of other regular physical exercises, and hours spent in other exercise modalities in a week. Classification of training status was based on the National Strength and Conditioning Association Guidelines (e.g., Beginner = 0–6 months; Intermediate = 8–12 months; and Advanced = over a year).

#### Physical activity readiness questionnaire (PAR-Q)

Participants completed the PAR-Q (Canadian Society for Exercise Physiology, n.d.) before beginning the program. The PAR-Q is a tool designed to assess an individual’s readiness for engaging in physical activity. It consists of a series of questions focused on identifying any potential health risks that could be exacerbated by increased physical activity. The questions address key areas such as cardiovascular health, joint and bone issues, and overall physical condition. Respondents answer “yes” or “no” to each question, with any “yes” responses indicating the need for further medical evaluation before participating in physical activities. The PAR-Q is valued for its simplicity, ease of administration, and effectiveness in ensuring the safety of individuals starting new exercise routines.

#### Attendance and retention

Coaches recorded attendance for every class. Participants that attended one, two, or three times a week represented the completion of 8, 16, or 24 possible sessions attended for one, 8-week program or 16, 32, 48 sessions over 16-weeks, respectively.

#### Self-efficacy

Self-efficacy was assessed using the Resistance Training Self-Efficacy scale (RT-SE) (Bandura, 1997; McAuley and Mihalko, 1998) to assess participants’ beliefs related to mastery experiences, physical capability, and resilience. The RT-SE scale considers a wider range of factors related to overall self-efficacy in resistance training, including exercise-specific confidence, belief in program adherence, managing fatigue, progressing in exercises, and overcoming barriers.

#### Psychological needs

The Psychological Needs Scale for Exercise (PNSE) is a fundamental tool for assessing individuals’ psychological experiences in the realm of exercise. It comprises three core components: competence, autonomy, and relatedness. Competence pertains to an individual’s confidence and perceived ability in executing exercises, overcoming challenges, and attaining fitness goals. Autonomy reflects one’s sense of control and freedom in designing exercise routines, setting goals, and aligning activities with personal preferences and values. Relatedness assesses feelings of connection, support, and understanding within exercise environments, encompassing social interactions, relationships with peers or trainers, and a sense of belonging.

#### Reasons to exercise (REX)

The Reasons to Exercise (RE_X_) Scale-2 was used to assess the reasons people exercise at baseline and post-program. The RE_X_-2 scale contains nine factors represented by 36 items including: (a) fitness; (b) competition; (c) solitude; (d) social; (e) appearance; (f) weight management; (g) health concerns; (h) mood enhancement; (i) preventative health. Items included a standardized stem (i.e., “To you, how important is this reason for exercising and/or being physically active?”) followed by content statements evaluated using a 6-point Likert scale, ranging from 1 to 6 (*not at all important* to *extremely important*). The validity and reliability of the RE_X_-2 demonstrated good psychometric properties in adult exercisers (Martinez Kercher et al., 2022).

#### Behavioral regulation

The Behavioral Regulation in Exercise Questionnaire-3 (BREQ-3) (Markland and Tobin, 2004) was used to assess the type of self-determined motivation ranging from amotivation to the most intrinsic form of motivation (i.e., relative autonomy) at baseline and post-program. The BREQ-3 measures external, introjected, integrated, identified, and intrinsic forms of regulation of exercise behavior using a 5-point Likert scale, ranging from 0 to 4 (*not true for me* to *very true for me*). The validity and reliability of the BREQ-3 has demonstrated good psychometric properties with adult exercisers (Markland and Tobin, 2004). In line with previous literature, a single score was computed by summing subscale scores to provide an index of the degree to which respondents felt self-determined to measure ‘relative autonomy index’ (RAI) (Martinez Kercher et al., 2022).

#### Resistance training intentions

Participants were asked to complete questions regarding intentions to continue RT in barbell. Participants’ intentions were assessed at Week 0, 8, and 17 using the following items: “Rate how likely you are to train in CE over the next 8 weeks;” “I intend to engage in barbell at least 2 times a week for the 8-week session;” and “I intend to engage in barbell at least 3 times a week for the 8-week session.” Each item has a Likert scale of 1 to 7, with anchors ranging from “Very unlikely” to “Very likely.” The two items will be analyzed individually.

#### Strength testing

Participants completed RT muscular strength and endurance testing at baseline and post-program. Exercises to assess muscular strength used a barbell and included the deadlift, front squat, and hang clean. For the muscular strength tests, participants completed a 3–5 repetition maximum set that was used to calculate a 1 rep max following NSCA guidelines (Table 1). Muscular endurance was assessed with a maximum push-up test. To account for differences in body mass, we calculated a relative strength index that summed participants total predicated 1RM loads for the deadlift, front squat, and hang clean then divided that sum by their body weight (e.g., [200 + 100 + 75]/170 lbs. = strength index).

**Table 1:** Power and strength-based compound barbell exercises used in programming.

**Complex power**	**Complex strength**
Hang clean*	Back squat
Power clean	Deadlift*
Snatch	Front squat*
Split Jerk	Bench press*
Hang High Pull	Split squat
Push press	Hex Bar Deadlift

*Exercises that will be included in the 3–5 RM (estimated 1 RM) testing to identify exercise intensity and workload.

### Procedure

Participants were asked to come to the medical fitness center on two separate occasions to collect measures before (Week 0) and after (Week 17) their 16-week barbell program. On the first visit, following a verbal and written explanation of the nature involved in the study, a written informed consent was obtained in accordance with the principal investigator’s Institutional Review Board. Once the participants provided informed consent, participants were asked to complete two questionnaires, the PAR-Q and a health history to determine the presence of contradictions to exercise, with a specific focus on orthopedic, cardiovascular, and pulmonary conditions that would preclude participation in the research study. Additionally, participants completed the PADRTQ.

On the first and last visit, the PADRTBQ, RT intentions, and questionnaires were collected from the participants. Following collection of psychological measures, participants’ anthropometrics and body composition were measured. During their first visit, participants underwent a familiarization session to teach them how to navigate through their workout sheet and where to report psychological measures, as well as how to record load (weight) lifted.

Participants were responsible for self-selecting their loads during lifts. However, it is a pseudo self-selected process because they were in a supervised setting where the coach may or may not encourage them to lift heavier or lighter. Generally, the coaches were encouraged to allow the participants to self-select, but through building the coaching relationship coaches often had the power to influence the weight lifted. We felt this supervised self-selected load approach would lead to an enhanced sense of autonomy within the lifter while utilizing the coach’s judgment and experience in the process.

### Data analysis

Data analysis was conducted using SPSS (Corp, 2024) and R Studio (2023.12.0 + 369) (RStudio Team, 2024). We conducted thorough data screening, including checks for missing data, outliers, and assessing univariate and multivariate normality. Internal reliability for each construct was measured using Cronbach’s alpha. Descriptive statistics were utilized with frequencies and percentages calculated for categorical variables and means along with standard deviations computed for continuous variables. Our analysis of outcome measures involved mixed model regressions and two-sample t-tests. Additionally, a sensitivity analysis was performed on participants who completed strength testing at both baseline and post-program to detect any systematic differences between these groups.

## Results

### Descriptive statistics

Table 2 shows the descriptive statistics for the final sample of adults (that completed the psychological measures) (*n* = 43). The sample was 100% white/Caucasian.

**Table 2:** Participant characteristics.

**Variable**	Male (*n* = 8)		Female (*n* = 35)	
Age	43.62 ± 10.47		45.42 ± 10.90	
Education (*n*%)				
Some high school	0%		2.80%	
Trade/vocational certification	12.50%		0%	
Some college credit	25%		2.80%	
Associate degree	0%		8.50%	
Bachelor’s degree	25%		42.80%	
Some graduate school	0		2.80%	
Master’s degree	37.50%		34.20%	
Doctorate degree	0%		5.70%	
Body composition (M, SD)	Pre-program	Post-program	Pre-program	Post-program
Skeletal Muscle Mass (SMM)	79.77 ± 15.89	64 ± 9.7	59.10 ± 6.02	61.85 ± 4.34
Body Mass Index (BMI)	24.95 ± 3.59	24.8 ± 1.2	26.0 ± 3.94	26.78 ± 4.64

### Strength testing

There were *n* = 27 participants who completed baseline strength testing and *n* = 29 who completed post-program strength testing. The sample who completed the post-program strength testing showed improvements in the amount of weight lifted (pounds) in their predicted 1RM for the front squat, hang clean, and deadlift. There were no significant changes in body weight, body fat mass, or skeletal muscle mass. For the strength index, which controls for participants’ body weight, there was an average increase of 0.21 (*SD* = 1.96).

### Resistance training (RT) intentions

At the start of the program, mean intention to continue to attend barbell for another 8-weeks at least 2 times and/or 3 times a week was 6.6 (±0.16) and 4.8 (±0.31) on a 7-point scale ranging from 1 to 7. After completing 16-weeks in the program, participants reported a mean intention to continue to attend barbell for another 8-weeks at least 2 times and/or 3 times a week was 6.2 (±0.29) and 4.4 (±0.43) on a 7-point scale. There was no significant change in intention to continue training RT (*p* > 0.05).

### Changes in psychological factors

The primary objective was to explore potential changes in psychological factors pre- to post-program. Welch two sample t-tests were conducted for all measures of interests (e.g., psychological needs, self-efficacy, BREQ-3). Table 3 shows all t-test results.

**Table 3:** Strength testing outcomes (pre-program *n* = 27, post-program *n* = 29).

Variable	Pre (Mean ± SD)	Post (Mean ± SD)	Change (Mean ± SD)
1 RM hang clean	99.28 ± 32.5	101.78 ± 31.83	10.00 ± 6.41
1 RM front squat	116.2 ± 39.1	121.37 ± 39.77	11.31 ± 13.10
1 RM deadlift	198.2 ± 50.9	197.24 ± 51.34	−0.26 ± 14.09
Max pushup reps	20.1 ± 21.6	24.04 ± 19.18	4.3 ± 8.11

Data presented as means in lbs. (SD); 1RM is a predicted 1 rep max from actual 3–5 rep max test.

Additionally, linear regressions were conducted to evaluate if attendance in the Competitive Edge program influenced changes seen in competence, identified regulation, mastery, and resilience. Total attendance did not significantly predict any of the significant psychological factors. Table 4 shows all regression results.

**Table 4:** *T*-test results for psychological factors pre vs post.

Psychological factor	Pre-mean	Post-mean	*t*-value	DF	*p*-value
Basic psychological needs					
Autonomy	5.34 ± 0.57	5.52 ± 0.57	1.356	79.37	0.178
Competence	5.06 ± 0.84	5.3 ± 0.73	1.479	79.83	0.017*
Relatedness	5.26 ± 0.74	5.33 ± 0.66	0.422	79.97	0.673
Behavioral regulation exercise questionnaire					
Identified regulation	3.49 ± 0.49	3.24 ± 0.53	−2.190	79.97	0.0313*
Amotivation	0.11 ± 0.35	0.11 ± 0.28	0.012	78.77	0.989
Intrinsic motivation	3.25 ± 0.66	3.26 ± 0.66	0.004	79.42	0.996
Integrated regulation	3 ± 0.90	3.07 ± 0.86	0.392	79.78	0.695
Introjected Regulation	2.36 ± 0.99	2.26 ± 0.87	−0.443	79.91	0.658
External regulation	0.43 ± 0.49	0.44 ± 0.50	−0.056	79.08	0.954
Self-efficacy					
SE-physical	7.72 ± 2.25	8.24 ± 1.61	1.215	76.10	0.227
SE-mastery	8.27 ± 1.96	8.89 ± 1.08	2.402	66.44	0.021*
SE-resilience	7.99 ± 1.93	8.61 ± 1.10	2.852	67.99	0.007*

### Psychological factors predicting RT outcomes

The secondary objective was to assess if baseline psychological factors predicted total muscular strength outcomes. Linear regressions were conducted between each psychological scale (e.g., BPN, BREQ, SE) and their subscales with changes in strength index. No significant results were seen. Additionally, we sought to assess if baseline psychological factors predicted muscular endurance (total number of push-ups performed) outcomes. Linear regressions were used to explore these relationships. No significant predictors emerged. Table 5 shows model results.

**Table 5:** Linear regression results for significant psychological factors.

Model	Coefficient	Std. error	*t*-value	*p*-value
BPN-competence	0.016	0.012	1.312	0.197
SE-mastery	0.019	0.018	1.020	0.314
BREQ-identified regulation	0.004	0.008	0.480	0.633
SE-resilience	0.011	0.019	0.599	0.552

Moreover, baseline composite scores of psychological factors were compared to each lift tested (e.g., deadlift, front squat, and hang clean). The analysis found that the baseline basic psychological needs score significantly predicted the change in one-rep max (1RM) deadlift (*β* = 3.64, *p* = 0.039). Table 6 shows all regression results.

**Table 6:** Linear regression results for psychological factors and change in strength.

Predictors	*F*-statistic	Adjusted *R*^2^	*P*-value
Muscular strength			
BPN subscales	0.884	−0.008	0.457
BREQ subscales	0.971	−0.004	0.458
SE subscales	0.745	−0.018	0.531
Muscular endurance			
BPN subscales	0.414	−0.171	0.746
BREQ subscales	0.578	−0.267	0.739
SE subscales	1.253	0.059	0.347

### Reasons to exercise

Lastly, the exploratory analysis sought to examine the RE_X_ measure. At the start of the program, participants’ top three reasons for exercising were for (1) preventative health (*M* = 5.3 ± 0.79), (2) mood enhancement (*M* = 5.1 ± 0.78), and (3) fitness (*M* = 5.1 ± 0.61). Competition (*M* = 2.5 ± 0.12) was the least important reason people had for participating in exercise. After 16-weeks, participants reported (1) mood enhancement (*M* = 5.1 ± 0.84), (2) preventative health (*M* = 5.1 ± 0.99), and (3) fitness (*M* = 5.1 ± 0.62) as their primary reasons for exercising. Competition remained the least important reason for exercising. We conducted two-sample *t*-tests to see if any subscales changed from pre- to post-program. No significant changes were seen. Table 7 shows all *t*-test results.

**Table 7:** Regression results for 1RM and baseline psychological factor scores.

Predictors	*F*-Statistic	Adjusted *R*^2^	*P*-value
Deadlift 1RM Δ			
Pre BPN-score	0.312	−0.051	0.585
Pre BREQ-score	5.209	0.231	0.039*
Pre SE-score	0.343	−0.049	0.567
Front squat 1RM Δ			
Pre BPN-score	0.312	−0.051	0.585
Pre BREQ-score	0.276	−0.054	0.607
Pre SE-score	4.309	0.191	0.058
Hang clean 1RM Δ			
Pre BPN-score	0.031	−0.074	0.856
Pre BREQ-score	0.577	−0.031	0.461
Pre SE-score	0.483	−0.038	0.498

Additionally, analyses were conducted to see if any baseline RE_X_ subscales predicted changes in muscular strength. No significant predictors were found. However, looking at muscular endurance, the subscale weight management (*β* = 10.016, *p* = 0.046) was a significant predictor of increases in endurance. Likewise, the subscale solitude had a positive relationship with muscular endurance (*β* = 6.792, *p* = 0.037). Table 8 shows all regression outcomes.

**Table 8:** RE_X_ subscale pre vs pos.

REx subscale	Pre-mean	Post-mean	*t*-value	DF	*p*-value
Social	3.92 ± 1.03	3.92 ± 1.06	−0.021	78.592	0.982
Weight management	4.24 ± 1.18	3.92 ± 1.21	−1.207	78.733	0.231
Health concerns	3.0 ± 1.28	3.18 ± 1.22	0.668	79.768	0.505
Appearance	4.24 ± 1.08	3.96 ± 1.20	−1.107	76.728	0.271
Mood enhancement	5.12 ± 0.78	5.13 ± 0.85	0.036	77.497	0.971
Solitude	3.66 ± 1.33	3.73 ± 1.32	0.211	79.273	0.833
Competition	2.48 ± 1.34	2.69 ± 1.48	0.669	76.970	0.505
Physical health	5.25 ± 0.79	5.11 ± 1.00	−0.695	72.041	0.488

Lastly, to explore the REx measure with changes in strength, we tested the baseline composite score was tested against changes in 1RM. The linear regression found that the composite REx score significantly predicted changes in hang clean strength (*β* = 1.146, *p* = 0.036). Table 9 shows all regression results for REx score and changes in strength (Table 10).

**Table 9:** Linear regression results for REx subscales and change in strength.

REx subscales	*β*	SE	*t*-value	*p*-value
Muscular strength, adjusted *R*^2^ = 0.008, *p* = 0.423				
Social	−0.301	0.327	−0.920	0.364
Weight management	−0.230	0.336	−0.686	0.497
Health concerns	0.550	0.283	1.944	0.060
Appearance	−0.071	0.381	−0.185	0.854
Mood enhancement	−0.130	0.573	−0.228	0.821
Solitude	0.159	0.313	0.509	0.613
Competition	0.255	0.270	0.947	0.350
Physical health	−0.162	0.496	−0.328	0.745
Muscular endurance, adjusted *R*^2^ = 0.634, *p* = 0.115				
Social	4.025	1.919	2.098	0.103
Weight management	10.016	3.505	2.858	0.046*
Health concerns	1.810	1.296	1.397	0.234
Appearance	−5.148	3.054	−1.686	0.167
Mood enhancement	−4.903	3.770	−1.301	0.263
Solitude	6.792	2.216	3.065	0.037*
Competition	1.880	1.453	1.294	0.265
Physical health	7.157	4.614	1.551	0.195

**Table 10:** Linear regression results for baseline REx score and change in strength.

Predictors	*F*-statistic	Adjusted *R*^2^	*P*-value
Pre-REx-score			
Deadlift 1RM Δ	2.818	0.114	0.117
Front squat 1RM Δ	0.381	−0.046	0.547
Hang clean 1 RM Δ	5.432	0.240	0.036*

## Discussion

While there has been a plethora of studies conducted assessing the relationship between psychological factors and aerobic training (Reed and Buck, 2009; DiLorenzo et al., 1999; King et al., 1989), there is a gap in our understanding of the association between psychological factors and participation in RT, and more specifically, barbell-based RT. The overarching goal of this study was to examine the relationship between basic psychological needs, behavioral regulation, self-efficacy, and a longitudinal barbell-based RT program for adults. The present study had 4 key findings. First, the basic psychological need of competence increased from baseline to post-program. Second, for behavioral regulation the identified regulation subscale, increased from baseline to post-program; and the behavioral regulation composite score of the BREQ-3 predicted muscular strength increases in the deadlift. Third, for the self-efficacy subscales, both mastery and resilience increased from baseline to post-program. Fourth, from an exploratory standpoint, the reasons to exercise scale predicted increases in muscular endurance and muscular power. Taken together, our data empirically support multiple positive relationships between psychological factors and longitudinal barbell-based RT programming for adults while calling for more exercise psychology research on barbell-based RT.

The first key finding was a significant increase in participants’ sense of competence from pre- to post-program. Competence is one of the three basic psychological needs posited by the psychological needs mini-theory of self-determination theory and is a critical component of sustaining intrinsic motivation and multiple positive behavioral and health outcomes (Ryan and Deci, 2000; Deci and Ryan, 1985), including exercise adherence and overall well-being. Many of the exercises included in the RT program are traditionally seen as complex, challenging, and intimidating, especially in a population with limited RT experience. Thus, increasing participants’ competence in these compound movements (e.g., deadlift, front squat, hang clean) is an important achievement. Studies of sport and exercise have found mixed results in improving basic psychological needs, as competence is a relatively stable construct that takes time to change (Ryan and Deci, 2000). Longitudinal studies have demonstrated increased competence pre- to post-program for aerobic training programs, but there is a strong need for more research on psychological responses to RT (Cavarretta et al., 2019; Goldfield et al., 2015). There have been mixed results about the association of RT exercises to competence and these studies are often of short duration, assessing single-joint or upper extremity RT exercises (e.g., leg curl, bicep curl, bench press), and are often in competitive athletes, younger populations, or populations with pre-existing health conditions (O’Dowd et al., 2022; Collins et al., 2019). However, to our knowledge, this is the first study to show an improvement in competence from pre- to post- program from a longitudinal barbell-based RT program for adults. The distinction between stationary machines and single joint exercises compared to barbell-based RT exercises is important because barbell-based RT may have added benefits, including but not limited to functional strength, balance, coordination, core activation, greater hormonal responses, and joint health (Paoli et al., 2017; Suchomel et al., 2018). An important aspect of the RT program in the present study worth highlighting is the role of perceived feedback as a source of competence. For instance, in line with psychological needs, individuals often judge their competence through different sources of motivation than they use to judge their performance ability (Ryan and Deci, 2000). In this RT program, participants received a variety of rich, intentional information about their progress, including coach feedback and an individualized tracking sheet to promote self-comparison and degree of strength performance improvement over time. This feedback may support self-awareness and a sense of accomplishment related to speed or ease of learning challenging new RT exercises, improvements in amount of effort exerted, and week-to-week improvement (Martin and Nikos, 2007). Therefore, self-determination highlights the role of feedback as a social factor that influences motivation and behavioral outcomes. While the barbell-based movements used in the present program are relatively challenging, this study provides evidence that adult participants enrolled in a 16-week RT program can significantly increase their sense of competence.

The second area of key findings was related to motivation through behavioral regulation. The identified regulation subscale of the BREQ-3 increased from baseline to post-program and the BREQ-3 composite score predicted muscular strength increases in the deadlift. Identified regulation, defined as identified benefits of exercise (e.g., “I value the benefits of exercise”), is an important improvement because it is associated with long-term exercise adherence (Teixeira et al., 2012). Compared to integrated regulation, identified regulation represents the lower limit of autonomous motivation in which participation is regulated by goal values or the importance of behavioral outcomes (Teixeira et al., 2012; Ryan and Deci, 2000). This finding is important, but may be related to characteristics of the sample in that the majority of the participants were likely in the action or maintenance stages of change as they have joined an RT program; thus, this finding may not have been representative in a population distributed across different stages of change. Literature supports the concept that people’s behavioral regulation generally becomes more intrinsic over time (Kwasnicka et al., 2016), but to our knowledge this is the first study to demonstrate increased intrinsic motivation in a longitudinal barbell-based RT program for adults. Taken together with the first finding of increased competence pre- to post-program, increased identified regulation suggests that a longitudinal RT program has many positive effects on psychological outcomes. With long-term exercise adherence and lifestyle benefits in mind, other RT programs may be well-served to target these psychological factors, in addition to the more traditionally sought after physiological outcomes (e.g., strength/physiological improvements).

Another key outcome related to behavioral regulation was that the BREQ-3 composite score predicted strength increases in deadlift, the most fundamental and emphasized barbell-based exercise in the current RT program. Deadlifts, or hip hinging, are a fundamental exercise to many other barbell-based movements (e.g., hang clean, power clean, hang snatch) and they also are, arguably, one of the most important functional movements for activities of daily living (e.g., bending over, picking things up off the ground). This finding further supports the notion of potentially focusing program design on not only on physiological improvements (e.g., strength gains), but also attempting to design RT programs to target intrinsic motivation, as defined within the behavioral regulation continuum identified within the BREQ-3.

The third key finding was an increase in self-efficacy subscales pre- to post-program, including resilience and mastery. Increases in resilience have been seen in other research, where exercise, broadly defined, served as a mediator for resilience during the COVID-19 pandemic (Lancaster and Callaghan, 2022). In terms of resilience, Lancaster and Callaghan (2022) findings indicated that those who exercised during the pandemic were able to increase their resilience versus those who were sedentary. Indeed, the effect of RT on psychological function are well-supported with empirical evidence demonstrating exercise ensures healthy brain functioning (Deslandes et al., 2009). Moreover, the increase in resilience is also seen in research exploring military training, where RT is prescribed not only for physical readiness, but for psychological preparation as well (Szivak and Kraemer, 2015). Expanding on existing literature, our study extends the observed improvements in resilience resulting from long-term barbell-based RT among adults. Our findings, in conjunction with previous research, highlight resistance training as a valuable tool not only for the general population but also for individuals undergoing specialized training, enabling them to better cope with life stressors. Additionally, our findings add to physical activity literature specifically highlighting that participation in a 16-week barbell-based RT program has an important association to changes in perceived feelings of self-efficacy among a group of adults who may have built up preconceived belief that resistance training leads to increased likelihood of injury. To combat that potential preconceived notion, in this study we found that adults reported a greater sense of efficacy in their ability to “execute a lift safely” and/or perform complex, barbell-based RT exercises without feeling like they will get injured. Future resistance training programs could prioritize resilience enhancement to offer greater advantages to participants both within and outside gym environments.

Similar to the resilience subscale, the mastery subscale showed a significant increase from pre- to post-program. The mastery subscale primarily captures individuals’ confidence in mastering new skills, indicating that participants felt more prepared and capable of learning and taking on unfamiliar tasks. This may be compared to research exploring self-efficacy in a RT context (Dionigi, 2007), which found that in healthy adults, RT offered enhanced perceptions of mastery which led to greater feelings of self-efficacy. The varied psychological improvements seen from pre- to post-program in the current study suggest substantial benefits from exercise aside from the clear physical benefits. These improvements in self-efficacy, combined with previously discussed findings of increased competence and identified regulation from pre- to post-program warrant further rigorous investigation in longitudinal barbell-based RT programming for regular adults.

The final key finding was the reasons to exercise (RE_X_) scale predicted increases in muscular endurance and muscular power. Specifically, two subscales, weight management and seeking solitude, were significant predictors of increased push-up repetitions seen post-program. Weight management’s influence on exercise has been explored previously, with females citing it as their primary motivation for engaging in fitness routines (Kim and Cho, 2013). Notably, this motivation tends to be more prevalent in female populations (Yang, 1994), which aligns with our study where 81% of participants were female. This prevalence likely contributed to the observed trend of weight management serving as a motivator for enhancing muscular endurance. Moreover, seeking solitude predicted gains in muscular endurance. There is extremely limited research of the relationship between pursuing solitude and RT, but this exploratory finding warrants further investigation. Those who are motivated by solitude may particularly benefit from barbell-based RT as it takes a high degree of focus to perform movements like the deadlift or front squat, in comparison to activities like jogging, cycling, or single joint machine-based exercises (e.g., leg extensions). Further, the RE_X_ composite score predicted muscular power (i.e., 1RM hang clean). Similar to previous research that found multiple valued reasons were associated with greater physical activity levels (Martinez Kercher et al., 2022), participants in the present study with a greater number of highly valued reasons for exercise were more likely to increase their hang clean performance. Further research exploring the specific effects of weight management, solitude, and reasons for resistance training could provide valuable insights into optimizing exercise interventions for both physical and psychological health outcomes.

In conclusion, barbell-based RT remains a relatively untapped exercise option compared to aerobic training (e.g., running, cycling) or stationary machines (e.g., leg extension, bicep curl) and our study provides evidence for important associations between psychological factors, RT programming, and RT outcomes. Moreover, these findings hold the potential to guide future research and practical applications within exercise science, especially in crafting programs that cater to the holistic well-being of individuals participating in RT. Notably, our study had a substantial representation of women in the sample. Consequently, the insights gleaned from this research could be particularly beneficial for women. Moving forward, there’s an opportunity for further work to develop targeted programs that can benefit women, who are often underrepresented in exercise science, gym settings, and research as whole, thereby fostering inclusivity and equity in society.

The present study must be interpreted within its limitations. First, there was a lack of generalizability from the study sample in that the population was primarily White, self-selected into the program (i.e., more motivated and financially stable than the regular population), and had the financial means to afford small group RT programming. Second, while this study was a pragmatic longitudinal assessment of an existing barbell-based RT program, the lack of a control group limits the internal validity of the findings and must be considered as the findings represent correlation rather than causation. Lastly, while also a strength of the study, the sample was made of primarily women, so the findings are not necessarily generalizable to men. Despite these limitations, this study adds relevant evidence emphasizing the importance of combining exercise psychology in exercise performance-based studies to help develop a more comprehensive understanding of human motivation in barbell-based RT contexts. After all, human performance exists within a psychological context as humans have important thoughts, feelings, and motivations that influence their behaviors.

## Data Availability

The raw data supporting the conclusions of this article will be made available by the authors, without undue reservation.
