# Corrigendum: Psychological needs, self-efficacy, motivation, and resistance training outcomes in a 16-week barbell training program for adults

**DOI:** 10.3389/fpsyg.2025.1581706

**Published:** 2025-04-04

**Authors:** Vanessa M. Martinez Kercher, Janette M. Watkins, Janelle M. Goss, Liam A. Phillips, Brad A. Roy, Kyler Blades, Dana Dobson, Kyle A. Kercher

**Affiliations:** ^1^Department of Health & Wellness Design, School of Public Health-Bloomington, Indiana University, Bloomington, IN, United States; ^2^Department of Kinesiology, School of Public Health-Bloomington, Indiana University, Bloomington, IN, United States; ^3^Program in Neuroscience, College of Arts and Sciences, Indiana University, Bloomington, IN, United States; ^4^United States Military Academy, Westpoint, NY, United States; ^5^Logan Health Medical Center, Kalispell, MT, United States; ^6^Blades Athletic Performance Academy, Kalispell, MT, United States

**Keywords:** strength training, affective responses, barbell training, women's health, inclusion

In the published article, “McAuley and Mihalko, 1998” was not listed as a citation in the article, and “Bandura, 1997” was not cited in the correct section. Additionally, “Jones et al. (2016)” was incorrectly cited and should be removed from the article.

A correction has been made to section *Methods, Measures, Self-efficacy* to include both “Bandura, 1997” and “McAuley and Mihalko, 1998”, and to remove “Jones et al. (2016)”. The corrected text is shown below.

“Self-efficacy was assessed using the Resistance Training Self-Efficacy scale (RT-SE) (Bandura, 1997; McAuley and Mihalko, 1998) to assess participants' beliefs related to mastery experiences, physical capability, and resilience. The RT-SE scale considers a wider range of factors related to overall self-efficacy in resistance training, including exercise-specific confidence, belief in program adherence, managing fatigue, progressing in exercises, and overcoming barriers.”

The authors apologize for these errors and state that they do not change the scientific conclusions of the article in any way. The original article has been updated.
